# An Effective 3D Shape Descriptor for Object Recognition with RGB-D Sensors

**DOI:** 10.3390/s17030451

**Published:** 2017-02-24

**Authors:** Zhong Liu, Changchen Zhao, Xingming Wu, Weihai Chen

**Affiliations:** School of Automation Science and Electrical Engineering, Beihang University, Beijing 100191, China; lzpro@126.com (Z.L.); wxmbuaa@163.com (X.W.); whchenbuaa@126.com (W.C.)

**Keywords:** shape descriptor, category recognition, instance recognition, RGB-D sensors

## Abstract

RGB-D sensors have been widely used in various areas of computer vision and graphics. A good descriptor will effectively improve the performance of operation. This article further analyzes the recognition performance of shape features extracted from multi-modality source data using RGB-D sensors. A hybrid shape descriptor is proposed as a representation of objects for recognition. We first extracted five 2D shape features from contour-based images and five 3D shape features over point cloud data to capture the global and local shape characteristics of an object. The recognition performance was tested for category recognition and instance recognition. Experimental results show that the proposed shape descriptor outperforms several common global-to-global shape descriptors and is comparable to some partial-to-global shape descriptors that achieved the best accuracies in category and instance recognition. Contribution of partial features and computational complexity were also analyzed. The results indicate that the proposed shape features are strong cues for object recognition and can be combined with other features to boost accuracy.

## 1. Introduction

In the field of computer vision, the last few decades have considered object recognition to be a fundamental task and is still an active research topic. As is widely known, deep learning technology and convolutional neural networks have been extensively developed so that a great number of tasks in computer vision, including object recognition, have seen dramatic improvements due to the advances of deep learning. In addition to the several well-established models such as LeNet-5 [1]; GoogLeNet [2]; R-CNN [3]; etc., more models with advanced structures are being proposed [4,5,6]. However, in some cases where both optic cameras and infrared cameras are available, deep learning technology may not be the best solution for object recognition due to the heavy computational load and unexpected issues when applying deep learning on both RGB and depth data [7]. It has been shown that the shape features play an important role for object recognition both in cognitive neuroscience [8,9] and computer vision [10,11], as they contain rich discriminative characteristics of an object which could be useful cues for recognition. With the help of RGB-D sensors, the data from multimodal sources provide much more cues for recognition than the plain RGB data. Moreover, RGB-D data have made the extraction of these cues more convenient because the increase of dimensionality from RGB to RGB-D data results in a more precise description of the real shape. The purpose of this paper is to exploit the ability of some shape features extracted from RGB-D sensors for object recognition.

Shape features have been studied for several decades for their capability to describe the shape of an object, and are widely used in areas such as content-based image retrieval [12]; computer graphics [13]; and image registration [14]. According to different feature extraction methods, shape features can be classified into three groups: (1) contour-based features, which are extracted from the contour of a shape; (2) region-based features, which are extracted from the whole region of a shape; (3) hybrid features, which are a combination of both contour-based and region-based features.

A large group of methods that use shape features for object recognition are based on shape matching [15,16,17]. The basic idea is straightforward. First, a set of different shapes is made, each of which is used as an exemplar representing each category. Second, for a query shape, correspondences of feature points are found by solving a correspondence problem between the query shape and each example in the set. Third, an aligning transform function is estimated using the corresponding points. Finally, the distance between the two shapes is computed and a decision is made according to the minimal distance. The major problem with this kind of method is its heavy computational complexity. Solving for both of the correspondences and the alignment transform function requires a large amount of computation that is very time consuming.

Another group of methods use shape features as a discriminative object representation for classification [10,11,14,18]. Some of these use shape features called the global-based shape descriptor, which capture the global property of an object. Others use a local-based shape descriptor that captures the local property. Typically, a shape descriptor is generated through two steps: (1) the feature extraction step that extracts a specific shape feature from an object; and (2) the descriptor quantization step that generates the final descriptor from the extracted features produced in the first step. The global-to-global shape descriptor means that the descriptor uses global descriptor in the descriptor extraction step while the partial-to-global shape descriptor means it uses local descriptor in the descriptor extraction step. There are also some features that are designed for specific use [18]. These approaches present some challenging issues. One of these issues is high dimensionality, as shape features usually have higher dimension, which leads to computational inefficiencies. The second issue is that they are all extracted from 2D images, which limits their application to a 3D environment. A few methods exist to deal with 3D cases [11,14]; however, they either use shape features for object discovery rather than object recognition [11], or the limited number of shape features used in 3D object recognition. More shape features that are defined over RGB-D images need to be evaluated in their ability for object recognition in 3D scenarios.

In this paper, we propose a hybrid shape descriptor for object recognition. We extracted five features from contour-based images and five features from the 3D point cloud data. The contour-based images were obtained by jointly using the color and depth image by some contour detection algorithms. The 3D point cloud data were obtained by projecting the RGB-D image into the 3D coordinates. These features are further concatenated into a 10-dimension vector as the representation of an object. This is called the hybrid shape descriptor because it combines both the color and depth image and also captures the global and local properties of a shape. Category and instance recognition was performed. The performance was evaluated in terms of average accuracy, precision-recall curves, and computational time. The results show that the proposed hybrid shape descriptor outperforms some commonly used features such as kernel-based shape features [19] and bounding box-based shape features [10].

The remainder of this article is organized as follows: related works are reviewed and the contributions of this paper are summarized in Section 2; Section 3 describes in further detail the proposed hybrid shape descriptor; Section 4 presents and discusses the experimental results in comparison with several state-of-the-art shape algorithms; Finally, our conclusions and future work are presented in Section 5. To avoid confusion in the following sections, we refer to shape feature as the individual shape measure, and the shape descriptor as the combination of the individual measures.

## 2. Related Work

Shape features can be used in various applications such as object retrieval [20] and computer-aided design [21]. Object recognition is also one of its major applications. In this section, several works that used shape features for object recognition are reviewed and the contribution of this article is summarized at the end.

Researchers have proposed various descriptors for 3D object recognition. The Point Feature Histograms (PFH) descriptor [22] characterizes the local geometry at a point in the point cloud. The Fast Point Feature Histograms (FPFH) [23] are a simplification of PFH that reduce the computational complexity. The Viewpoint Feature Histogram (VFH) [24] adds viewpoint information to the FPFH descriptor.

Numerous methods have used shape features as a contributing factor, together with other characters, to represent an object. Lai et al. [10] combined shape features and visual features. Spin images from the shape retrieval community were utilized as shape features for classification. These spin images were finally represented by a 2703 dimension shape descriptor with the help of efficient matching kernel [25]. Han et al. [26] employed shape and appearance features for facial expression recognition. They extracted the FACS model—a coding system used to classify facial expressions according to the facial action [27]—to capture the shape of a face. Shape features can also be used together with local texture to recognize the interior photoelectric devices [28]. However, the dimension of features used in these methods is usually high and the shape features have to be combined with other features to get satisfactory results.

Liang and Juang [18] proposed an integrated system for the segmentation and classification of four moving objects. The image was first segmented into regions that potentially contained the target object. These regions were then transformed into Haar wavelet space and the local shape feature (i.e., the HOG descriptor in the wavelet space) was extracted from the space, resulting in the 1680 dimension shape descriptor. However, these features were specially designed for the classification of a few invariant objects, including pedestrians; cars; motorcycles; and bicycles. Ning et al. [29] introduced an approach for understanding the primitive shape of the scene to reveal the semantic scene shape structure and represent the scene using shape elements. Four shape features were defined: planar shape; cylindrical shape; spherical shape; and conical shape. These were used to represent 3D scene objects and differentiate them. However, this approach was only effective for a few types of objects where their shapes were regular and easy to differentiate, for instance, the ground, wall, windows, and doors. Hernández et al. [30] developed a vision system for the detection and location of the objects in indoor environments. Geometric shape descriptors and bag of words are implemented as two alternatives to extract features of the objects. This work is effective in detecting three objects present in indoor environments: chairs, closets and screens.

Karpathy et al. [11] introduced some common shape features to exploit their ability in object discovery, thus finding objects in 3D cluttered scenes. These features were extracted from point clouds and contributed to the ”objectness”, an index distinguishing objects from clutter. However, these features were used for object discovery, and the potential use of these features in object recognition needs to be exploited. As’ari et al. [14] managed to extract some shape features from depth images or 3D point clouds for object recognition. In this study, four shape features were extracted: shape distribution; local spin image; global spin image; and shape histogram; however, the number of features used in object recognition was limited. Additional shape features with discriminative ability need to be further examined.

In comparison with the above-mentioned approaches, the major contributions of our work are summarized as follows:

(1) This study exploits the ability of popular shape features previously used for other purposes for object recognition. In order to have a strong discriminative power, as most other features used in object recognition, the shape features used were chosen to capture both local and global characteristics of a shape.
(2) These features were concatenated in a simple way, resulting in a 10 dimensional vector as the final descriptor. The low-dimensional descriptor makes for effective computation and the further incorporation of other features.

## 3. Shape Feature Analysis

Researchers in psychology have revealed the effect of the shape features of an object (e.g., contours, symmetry, parallelism, etc.) on human vision perception [31]. These ideas were adopted in the field of computer vision by analyzing shape features in an attempt to improve object recognition accuracy. We first introduce five 2D shape measures, and then describe how the 3D measures defined in [11] were used in our algorithm. 2D measures were extracted from the color image while 3D measures were from the point cloud.

### 3.1. Algorithm Overview

**Figure 1:**
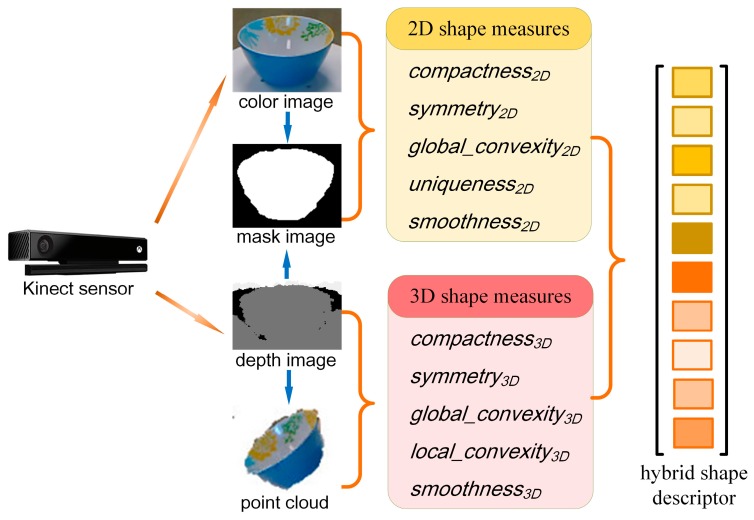
The flowchart of the proposed approach.

The flowchart of the proposed approach is summarized in Figure 1. A Kinect RGB-D sensor was used to obtain the color and depth image. Given the RGB-D images, objects need to be segmented out of the background. The algorithm combines the depth-based and vision-based segmentation methods which use visual cues, depth cues, and rough knowledge of the object-background configuration to produce a mask image. The 3D point cloud data are obtained using the RGB-D images covered by the mask image. For a detailed description of how the mask image and the point cloud data are obtained please refer to [10]. Using these data sources, we obtained the 10 shape measures described in the following subsections. Hence, the hybrid shape descriptor is obtained by concatenating the 10 shape measures for classification.

### 3.2. 2D Shape Measure

**Figure 2:**
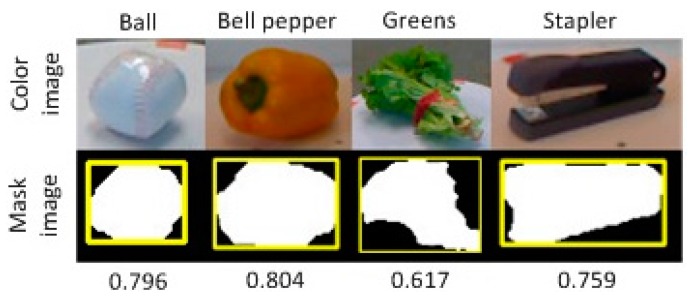
Compactness is measured by the ratio of the object area to the area of its smallest bounding box. The first row is the color image of each object. The second row is the mask image indicating the location of the object in the color image. The bounding box is highlighted in yellow.

Compactness represents the degree to which an object shape is compact. There exist several compactness measures, which are independent of scale and orientation, and not overly dependent on one or two extreme points in the shape. A common compactness measure, Isoperimetric quotient, is defined as the ratio of the area of the shape to the area of a circle having the same perimeter. For simplicity, we defined the compactness measure as the ratio of the area of the object to the area of its smallest rectangular bounding box.

(1) $${\mathrm{compactness}}_{2D}=\frac{\mathrm{area}\left(O\right)}{\mathrm{area}\left(B\right)}$$

where area(∙) computes the area of the shape. In the 2D case, it counts the number of pixels within the corresponding area. O denotes the object area in the mask image where pixel intensity is 1, and B denotes the corresponding bounding box of the object. Figure 2 illustrates the computation procedure.

Various researchers have addressed the crucial role of object symmetry in the visual perception system, both in psychology [8] and computer vision [32]. Various symmetry features have been defined. Sun [33] used symmetry as a high level feature in region growing image segmentation and region-of-interest (ROI) detection in brain magnetic resonance imaging (MRI) sequences. Huebner et al. [34] detected regional symmetry-based features that were sparse and highly robust to scale change in Panoramic Robot Vision Systems. Hauagge et al. [32] proposed a new technique for extracting local features from images of architectural scenes used for feature detection purposes and for computing descriptors. All of the above features were either high-dimensional vectors that are not suitable for describing a simple symmetry measure, or local features that cannot capture the whole symmetry property of an object.

We propose a simple but effective symmetry measure by computing the ratio of the overlap area of original and reflected image to the area of the original image. Specifically, we calculated our symmetry measure of an object using the following equation:

(2) $${\mathrm{symmetry}}_{2D}=\underset{i\in \left\{1,2\right\}}{\max}\frac{O\left(O,O_{-a_{i}},a_{i}\right)}{\mathrm{area}\left(Obj\right)}$$

where $O\left(O,O_{-a_{i}},a_{i}\right)$ is the overlap area of *O* and its reflection *O_−_a_i_* around axis *a_i_*. Since the image was captured by putting the object on a turntable, the layout of the symmetry axis of the same object was not uniform in images. To capture the symmetry measure of the same object in these images, we selected two symmetry axes, the vertical and horizontal symmetry axis of the mask image, as the symmetry axis hypothesis of the object. For each axis we computed a symmetry measure and then chose the maximum as the object symmetry measure.

**Figure 3:**
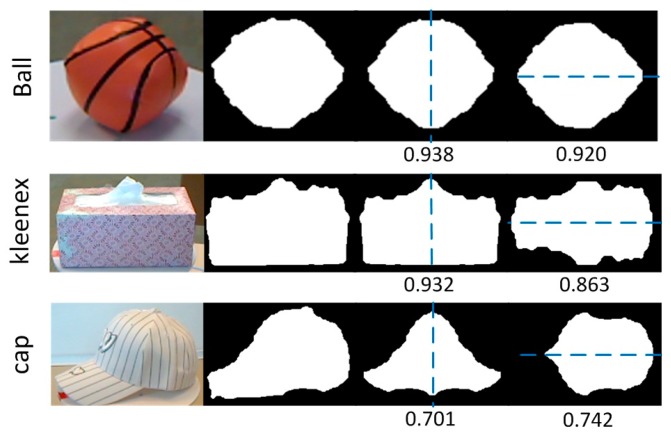
The symmetry score is computed as the ratio of the overlap area of original and reflected image to the area of the original image. Three categories are illustrated. Images in columns from left to right are: color images; mask images; overlap images along vertical axis; and overlap images along horizontal axis. The symmetry scores are labeled below.

Figure 3 illustrates how the symmetry measure was computed. It was observed that for a symmetrical object along two axes (e.g., the ball), both of the scores were high. For an object that was symmetrical along a single axis (e.g., the Kleenex), one of the two measures was relatively low, but the final symmetry score remained high. For an asymmetrical object (e.g., the cap), both of the measures were low, as was the final score. Therefore, it can be said that the symmetry measure is an effective indicator of whether an object is symmetrical.

**Figure 4:**
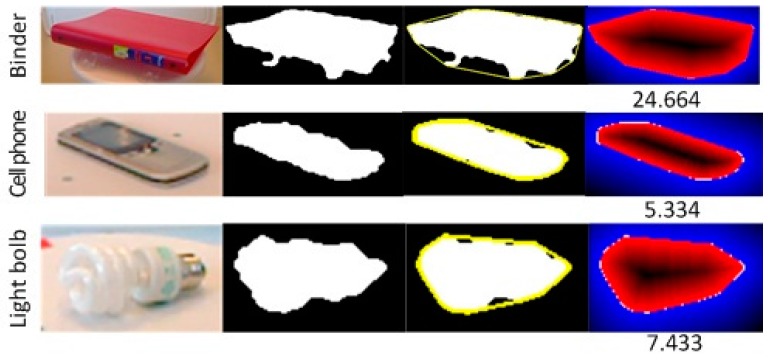
Global convexity is measured as the average distance of the points from an object to its convex hull. Three categories are illustrated. Images in columns from left to right are: color images; mask images; the convex hull; and the distance of points to the convex hull. The convex hull is highlighted in yellow. The different distances are colored in different colors and the global convexity scores are labeled below.

Another property of an object’s shape is global convexity, where a set of points is convex if (a) every internal angle is less than or equal to 180°; and (b) every line segment between two points remains inside or on the boundary of the set. In mathematics, the convex hull of a set *X* of points in the Euclidean space is the smallest convex set that contains *X*. The more a shape is convex, the more points it has on the boundary of its convex hull. In this paper, we define the object global convexity measure as the average minimum distance of the points inside an object to its convex hull,

(3) $$\mathrm{global}\_{\mathrm{convexity}}_{2D}=\frac{1}{N}\overset{N}{\underset{1}{\sum }}\underset{j}{\min}\mathrm{dist}\left(O_{i},h_{j}\right)$$

where *O_i_* denotes the *i*-th pixel in Object *O*; *N* is the total number of pixels in the object; *h_j_* denotes the *j*-th pixel of the object’s convex hull; and dist(∙,∙) denotes the Euclidean distance between two points. Examples can be seen in Figure 4.

The contour of an object contains necessary intrinsic information as we can sometimes recognize an object just by its contour. In order to capture this information, uniqueness and smoothness was used. They are computed based on the tangent angle histogram, which is computed in two steps: (1) compute the tangent vector for each point on the contour; and (2) compute the histogram of the angle difference of these tangent vectors. To obtain the tangent vector of each point on the contour, inspired by the first-order derivative of the univariate function, we attempt to compute the difference of two adjacent points,

(4) $$v_{i+1,i}=\left(x_{i+1},y_{i+1}\right)-\left(x_{i},y_{i}\right)$$

where ($x_{i},y_{i}$) denotes the location of point *i*, and $v_{i+1,i}$ represents the tangent vector from point *i* + 1 to point *i*. However, we found that the tangent vector $v_{i+1,i}$ remained in only four fixed directions, namely, horizontal; vertical and 45° to horizontal. To accurately reflect the actual direction, we obtained the final tangent vector of each point by computing the difference of its four neighborhoods,

(5) $$v_{i}=v_{i+2,i+1}-v_{i-1,i-2}$$

**Figure 5:**
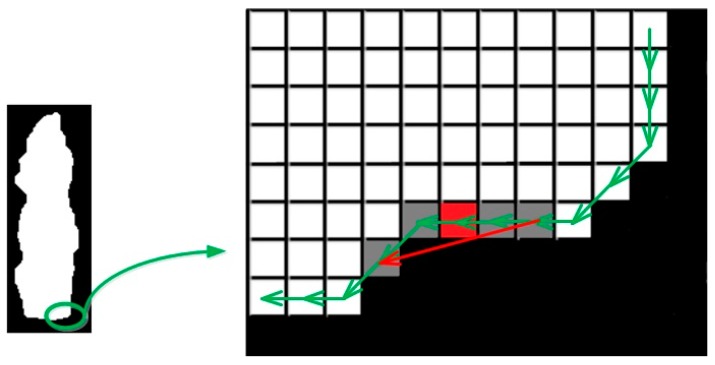
Computation of the contour point’s tangent vector based on four neighborhoods. For example, the tangent vector of the point in red is computed as the summation of vectors of its four neighborhoods in gray.

Figure 5 illustrates the detailed computation procedure. Next, the angle between two tangent vectors need to be computed. The choice of the two points could be either adjacent (between point i and *i* + 1) or separated by several points (between point *i* and *i* + *t*, *t* > 1). The choice of t will have an impact on the distribution, and thus, will have an impact on the uniqueness and smoothness measure. In the experiments, t was set empirically to be 3. A histogram of those angles ranging from zero to 180° is obtained. The smoothness of the contour was represented by the distribution of the angle. A distribution centered around the lower angle region indicated a smoother contour surface, whereas more values distributed around the greater angle region represented a rougher contour surface. Uniqueness was defined as the entropy of the histogram to evaluate the distribution of the tangent vector angles.

(6) $${\mathrm{uniqueness}}_{2D}=-\overset{n}{\underset{i=1}{\sum }}h\left(i\right)\log\;h\left(i\right)$$

where *h*(*i*) is the histogram value in the *i*-th bin; *n* is the number of bins. Therefore, if the entropy is small, the distribution is unique and is more likely to contain a single kind of angle, either acute or obtuse; if the entropy is large, the distribution is more complicated and it is more likely to contain both acute and obtuse angles.

We also observed that for most cases, the distribution had two local peak values. One was greatly centered around the lower angle region, the other was smaller centered around the higher angle region. This inspired us to define another measure: smoothness. Thus, we fit a Gaussian Mixture Model (*k* = two) to the histogram and assigned the mean of the Gaussian Model with a higher value to smoothness,

(7) $${\mathrm{smoothness}}_{2D}=\max\;\left(\mu_{1},\mu_{2},\ldots ,\mu_{k}\right)$$

where $\mu_{1}$ is the mean of the *i*-th Gaussian variable.

**Figure 6:**
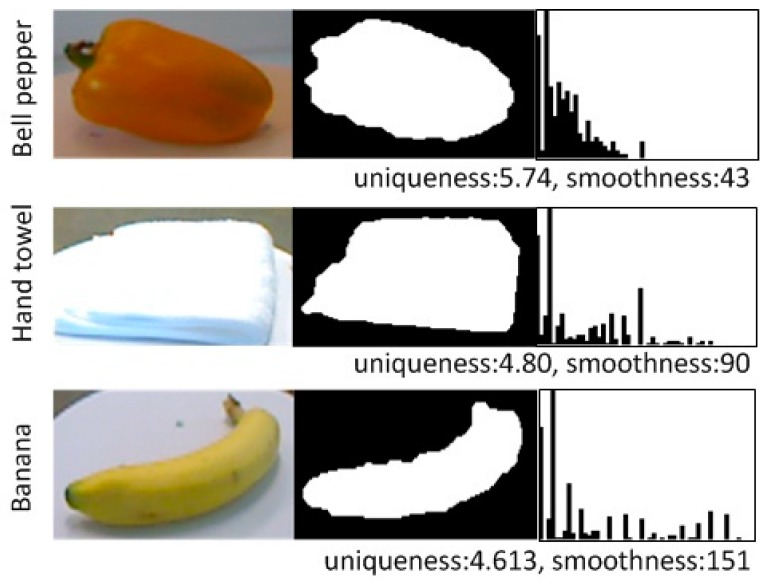
Uniqueness and smoothness are two numeric characteristics of this histogram. Images in columns from (**left**) to (**right**) are: color images; mask images; and the histograms of the angle difference of tangent vectors. The histogram ranges from zero degrees to 180° from left to right. Three categories were analyzed and the values of these two measures for each category are listed below.

Figure 6 analyzes the histogram of the tangent vector angle difference for the selected categories. For the bell pepper category, the contour changed slightly and the angle difference between the two adjacent tangent vectors was small. This notion is reflected in the histogram image, where one can see that most of the angles are distributed in the lower angle region. For the hand towel category, the contour mainly consisted of two types; straight lines and right angles. This is also reflected in the histogram, where angles are mainly centered around zero degrees (left) and the 90° region (center). For the banana category, the contour consisted of smooth curves and sharp angles, hence the distribution was mainly centered at the lower angle (left) and the higher angle regions (right).

### 3.3. 3D Shape Measure

In order to conduct 3D shape measure, we extracted five common shape features over point clouds. The task of extracting features that were originally defined on color images over point clouds was not an easy process. Karpathy et al. [11] implemented some of these features based on segmented scene meshes for the task of general object discovery. The scenes in 3D mesh format were taken as input data, and divided into a large collection of segments. Six shape features were extracted over each segment and combined as the overall objectness measure. The segment that scored the top objectness was regarded as an object. Inspired by this work, our study adopted five of those shape features for extraction over point clouds.

The point cloud data are stored in *x-y-z* format, with *p*(*x*, *y*, *z*) representing the coordinates of the point in the 3D world. Then Principal Component Analysis (PCA) is applied to the set of points in a segment of point cloud. As we all know that applying PCA on the 3-dimensional data will produce 3 principal axes. *p*(*x*, *y*, *z*) is projected onto these principal axes to get the new coordinates. The aim of applying PCA to the point cloud is to get a more uniform representation of segment. Otherwise, the coordinates of two point clouds with the same shape might differ a lot due to the different choices of the origin in the world coordinate. Moreover, normal vector is estimated for each point by selecting the smallest principal axis of the PCA on a set of neighboring points. With the new coordinates and the normal vector for each point, the following five 3D shape measures are extracted.

Compactness aims to measure the degree to which the object occupies the bounding box, where the bounding box of a segment of point cloud is the smallest cubic box that enclosing the points. Compactness was defined as the ratio of the total surface area of the cloud C to the surface area of its bounding box B,

(8) $${\mathrm{compactness}}_{3D}=\frac{\mathrm{area}\left(C\right)}{\mathrm{area}\left(B\right)}$$

However, it is computational inefficient to compute the surface area of a point cloud. Instead, we used two alternatives to represent area(C) and area(B), respectively. area(C) is computed as the number of points in the cloud. To compute area(B), the length (*x*-axis), width (*y*-axis), and height (*z*-axis) of the box need to be known. Here, only the length is used, i.e., length^2^.

As stated earlier, symmetry plays an important role in both human visual perception and computer vision. We computed this feature by summing up the overlap area between the original cloud *C* and its reflection *C_−d_* along three principal axes *r_d_*.

(9) $${\mathrm{symmetry}}_{3D}=\underset{d\in \left\{x,y,z\right\}}{\sum }\frac{\lambda_{d}}{\Lambda }\left[O\left(C,C_{-d},r_{d}\right)+O\left(C_{-d},C,r_{d}\right)\right]$$

where $\Lambda =\lambda_{x}+\lambda_{y}+\lambda_{z}$, and $\lambda_{d}\left(d\in \left\{x,y,z\right\}\right)$ denotes three eigenvalues of the cloud.

Global convexity evaluated the degree to which an object’s convex hull is an approximation to the object’s shape. The convex hull of a set of points is the smallest convex set that contains these points. The global convexity was computed as the average distances from a point in the cloud to the closest point on the convex hull.

(10) $$\mathrm{global}\_{\mathrm{convexity}}_{3D}=\frac{1}{N}\overset{N}{\underset{i=1}{\sum }}\underset{j}{\min}\mathrm{dist}\left(C_{i},H_{j}\right)$$

where *C_i_* denotes the *i*-th point in cloud *C*; *N* is the total number of pixels in the object; and *H_j_* denotes the *j*-th point of the cloud’s convex hull. In practice, the convex hull of a point cloud is obtained by the function provided by the Point Cloud Library.

In real-world scenes, the Kinect sensor usually sees only part of the object. The obtained 3D cloud contains points lying on the visible portion of the object surface. Such a cloud might become a concave object. However, even if the cloud is concave, the convex hull is always convex. For example, when Kinect sensor sees half of a basketball, the cloud is a hemisphere. The convex hull of it is closed and convex. The global convexity measure is still valid. An exception occurs when the object itself is concave. For example, if we see a bow from the top-down view and from the bottom-up view respectively, the two situations will give the same global convexity results. In this case, the global convexity cannot tell whether the object is convex or concave. However, the probability that this happens is very small because that we usually see the objects in a single view, and that we seldom see two objects with the same shape but one of them is convex and the other is concave.

Surfaces of objects are often made up of locally convex regions, e.g., the handle of a mug or the wheel of a mouse. Local convexity is used to analyze this surface property. Let $P={\left\{p_{i}\right\}}_{i=1}^{N}$ be the *N* points contained in a point cloud. We compute the neighborhood points for each $p_{i}$ within a certain radius, denoted by $P_{i}={\left\{p_{j}\right\}}_{j=1}^{N_{i}}$. We use the following rule to determine point $p_{j}$ to be relatively convex to point $p_{i}$:

$$n_{i}^{T}\cdot \frac{p_{j}-p_{i}}{{\left|\left|p_{j}-p_{i}\right|\right|}_{2}}<0$$

where $n_{i}$ denotes the normal vector at point $p_{i}$. We count the number of points in $P_{i}$ that are convex to point $p_{i}$, denoted by $n_{i}$, and divided by the total number of points in $P_{i}$ to obtain the score $s_{i}=n_{i}/N_{i}$. The local convexity is computed by averaging the score of all $s_{i}$:

$$\mathrm{local}\_{\mathrm{convexity}}_{3D}=\frac{1}{N}\overset{N}{\underset{i=1}{\sum }}s_{i}$$

Smoothness measures an assumption that the mass of a cloud should be uniformly distributed around each point. The algorithm first projects a point and its neighborhood to the tangent plane defined by its normal, then converts the angle of points into a histogram, the entropy of which is a smoothness score of the point.

In practice, we extracted the 3D shape measures based on the method proposed by Karpathy et al. [11], and their implementation details for extracting these measures is specified in their paper.

### 3.4. Computational Complexity Analysis

The computational load mainly comes from two aspects: the feature extraction phase where 10 shape features are extracted and the classification phase where the parameters of the classifier are estimated.

In the feature extraction phase, the complexity of extracting 2D shape features can be ignored compared to the 3D feature extraction, which needs 3D point cloud processing. The most time consuming step is the surface normal estimation, which consists of K-Nearest Neighbor (KNN) search, Random Sample Consensus (RANSAC) surface fitting, and PCA. Let l be the descriptor dimension, n be the total number of points in the cloud. For KNN search, it requires O(l) operations to compute the distance between two points, O(nl) operations to find one nearest neighbor, and thus the overall complexity is O(knl) to find k nearest points. For PCA, the covariance matrix computation needs $O\left(l^{2}n\right)$ operations, the eigenvalue decomposition needs $O\left(l^{3}\right)$ operations. So, the complexity of PCA is $O\left(l^{2}n+l^{3}\right)$. The computation of complexity of RANSAC algorithm is rather complicated because it is an iterative method and also depends on many parameters. The complexity of the proposed descriptor is almost the same as most existing descriptors because most of them are dominated by surface normal estimation.

In the classification phase, the advantage of the proposed descriptor over existing descriptors lies in this phase because the dimension of the proposed descriptor is relatively low. Taking SVM for example, let $N_{s}$ be the number of support vectors and consider the case $N_{s}\ll n$. The complexity of SVM in the training phase is $O\left(N_{S}^{3}+N_{S}^{2}n+N_{s}ln\right)$ [35]. Existing descriptors usually have dimension l ranging from $10^{3}$ (kernel PCA based shape features (KPCA), spin kernel descriptors (Spin KDES), and the shape features (SF)) to nearly $10^{4}$ (depth kernel descriptors [19]). In such cases, l≈n. The complexity becomes $O\left(N_{S}^{3}+N_{S}^{2}n+N_{s}l^{2}\right)$ in which l becomes a dominating parameter. Hence, the proposed descriptor allows a fast SVM classification.

## 4. Experiment Results

In this section, we tested the performance of the proposed shape descriptor for the task of object recognition using the UW RGB-D Object Dataset [10]. This dataset contains 250,000 RGB-D images of 300 common everyday objects captured from multiple view angles. The images are organized in category structure using WordNet hyponym/hypernym relations. There are 51 categories in the dataset, which are organized in alphabetical order from apple to water bottle. Each category consists of several instances, and each instance consists of multiple view images where the viewing angles are 30°, 45°, and 60° with the horizon.

The goal of object recognition is to assign a class label to each query image. This is achieved by training a classifier in training examples with corresponding predefined class labels. We evaluated our descriptor at two levels: category recognition and instance recognition, which are two basic capabilities for vision-based service robots, manipulators and surveillance systems. The notion of category and instance recognition can be illustrated as follows: a robot must have the ability to distinguish mugs from other categories like a note book or a stapler; it should also be able recognize between which is Peter’s and which is Mary’s. The former is considered category recognition, otherwise known as inter-class recognition, and the latter is known as instance recognition, or intra-class recognition.

We followed the experimental setup proposed by Lai et al. [10], and compared the proposed shape descriptor with several popular shape descriptors in the following subsections.

### 4.1. Comparison with Common Global-Based Shape Descriptors

#### 4.1.1. Experimental Setup

Two levels of recognition performance were evaluated. For category recognition, we randomly removed one object from each category for testing and trained the classifiers on the remaining 299 objects. For instance recognition, we took the image sequence at 30° and 60° with the horizon for training and left the ones at the angle of 45° for testing. Hence, category recognition meant recognizing previously unseen objects as belonging to a certain category from the training objects, while instance recognition meant determining whether an object was physically the same object as had previously been seen. We took every tenth video frame from the turntable data, and resulted in sampling 21,033 point clouds and contour-based images.

The recognition performance was evaluated using four category-specific classifiers: Naive Bayes (NB); Nearest Neighbor (NN); linear support vector machine (LinSVM); and Gaussian kernel support vector machine (kSVM). For SVM classifiers, we treated the problem as a multi-class classification. To determine parameters when using SVM classification, we set a range of values and trained a classifier model with each combination of these values. Finally, we chose parameter combinations that scored the best average precision. In each loop of training a classifier, we performed five fold cross validation. We randomly split the training set into two parts, leaving out 20% of the training examples for cross validation, and used the other 80% as training examples. For category recognition, we repeated this procedure a total of five times. The final accuracy is the mean of average precisions for all times. For instance recognition, this was onlydone once.

We compared the proposed descriptor with three common shape descriptors in the object retrieval community: shape distribution [36]; global spin image [37]; and shape histogram [38]. First, we needed to extract those descriptors over point clouds obtained from the Kinect-like sensor. In terms of shape distribution (SD), first, we randomly selected 1024 × 512 pairs of points from each point cloud. Next, we computed the Euclidean distance between every pair of points from which we obtained the minimum and maximum distance. The interval between the two values was discretized into 1024 bins where the value of each bin corresponded to the number of pairs of points whose distance values just fall into the bin. The result was a 1D histogram, which can be regarded as the descriptor of the point cloud. Global spin image (GSI) generates only one spin image to represent a point cloud. The centroid point is selected as the oriented point. The width of spin image was set to 40, the bin size was two millimeters and the support angle was defined as 60°. The result was a 1600 dimensional array, which was a 1D histogram. In shape histogram (SH), we used 100 shells to segment each point cloud. The centroid point of the point cloud was selected as the common center of these shells. First, we computed the longest distance one, from the centroid point to other points in the point cloud. Then, we divided one by 100, which was set as the dimension of the feature and generated an r value. Finally, we inferred that the radius of each shell increased with r and a 100 dimensional 1D histogram was produced as the shape feature.

#### 4.1.2. Results

After extracting features, we trained the above-mentioned four classifiers at levels of category recognition and instance recognition based on the UW RGB-D Object Dataset. Table 1 reports accuracies of the proposed hybrid shape descriptor in comparison with three aforementioned global-based shape descriptors. The results show that our work consistently outperforms other shape features in category recognition by an average of 18.4%. For instance recognition, the proposed descriptor achieved better accuracy with three classifiers, with the exception of the nearest neighbor classifier.

### 4.2. Comparison with Some Local-Based Shape Descriptors

#### 4.2.1. Experimental Setup

In this section, we compared the proposed shape descriptor with local-based shape descriptors [10,19]. In order to compare our results with those descriptors, we employed the same experimental setup suggested by [10]. This setup was almost the same as that described in Section 4.1.1, the only difference being that we took every fifth video frame from the turntable data, which resulted in sampling 41,877 point clouds together with contour-based images.

#### 4.2.2. Results

In Table 2, we compared our shape descriptor with kernel PCA based shape features (KPCA) and spin kernel descriptors (Spin KDES) proposed by [19], and the shape features (SF) that combines spin images and 3D bounding boxes used in [10] with linear support vector machine (LinSVM) and Gaussian kernel support vector machine (kSVM), respectively. The results show that the proposed descriptor is comparable to the shape descriptors that achieve the best accuracies both in category and instance recognition, with slightly better accuracy (by 1.6%) in category recognition and lightly lower accuracy (by 0.4%) in instance recognition. It is worth noting that the partial-to-global descriptors usually have more discriminative power than global-to-global descriptors. Moreover, the proposed descriptor has a low dimension compared with other four descriptors (1000 dimension for KPCA and Spin KDES; 2703 dimension for SF).

### 4.3. Contributions of Partial Features

**Figure 7:**
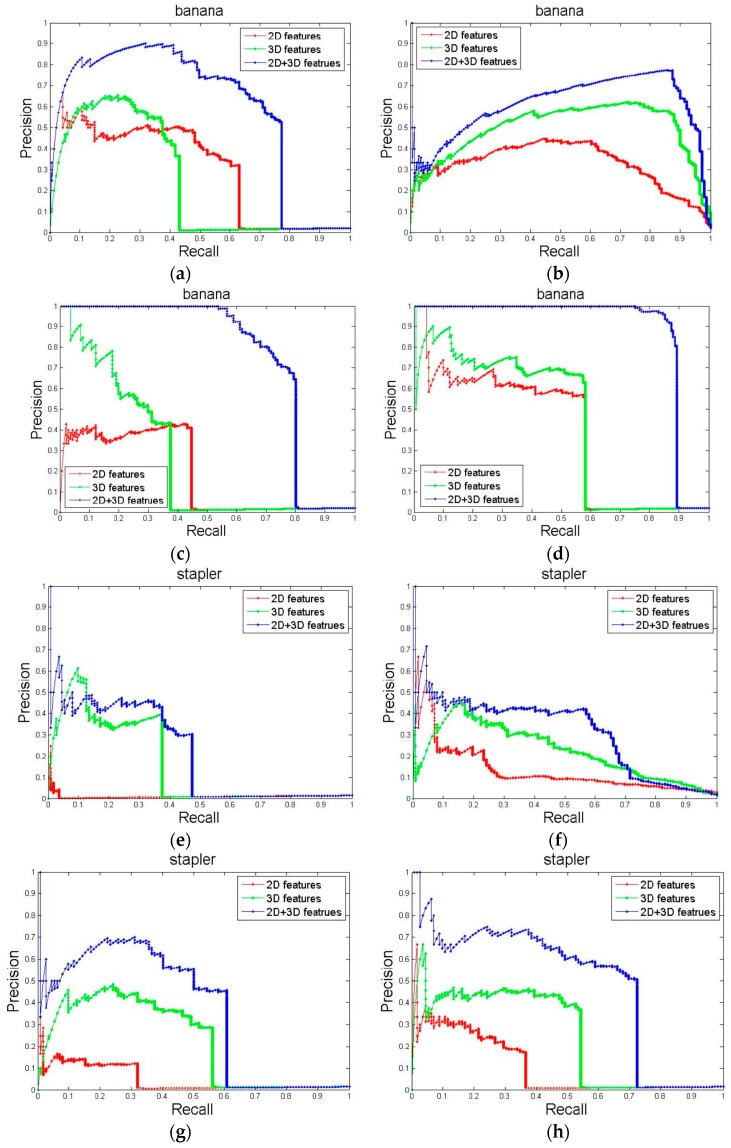
Precision-recall curves comparing performance with 2D shape features only (red), 3D shape features only (green), and both (blue) using 4 different classifiers. (**a**) Result of banana category using NB classifier; (**b**) Result of banana category using NN classifier; (**c**) Result of banana category using LinSVM classifier; (**d**) Result of banana category using kSVM classifier; (**e**) Result of stapler category using NB classifier; (**f**) Result of stapler category using NN classifier; (**g**) Result of stapler category using LinSVM classifier; (**h**) Result of stapler category using kSVM classifier.

We analyzed the contribution of only 2D features, only 3D features, and all ten shape features to the above four classifiers using the same process. Figure 7 lists the precision-recall curves of classification in the category of banana and stapler. From this, we can draw two conclusions. First, the classifiers that achieve average precision basically, in ascending order are: Naive Bayes; Nearest Neighbor; Linear SVM; and Kernel SVM. Second, the average precision of only 2D features was less than that of only 3D features, and all features achieved the highest average precision.

### 4.4. Computational Time Comparisons

The computational time was compared with respect to two primary phases: the feature extraction phase and the classifier training and testing phase. The first instance of the first category in the UW RGB-D Object Dataset (“apple_1_1_1”) was used for the experiment. We measured for each algorithm the processing time per frame and the results were averaged over 100 frames. The average number of points of these 100 point clouds are 3361. The feature extraction code was written in C++ based on Point Cloud Library, the classifier training code was written in Matlab. We used a PC with Intel i7-3770 CPU and 16GB RAM.

#### 4.4.1. Feature Extraction Phase

Recall that, prior to extracting the specific shape descriptor, two steps are necessary: estimating the surface normal vector and projecting the point cloud to its eigenbasis using PCA. We measured these two steps together with the remaining steps that completes the feature extraction phase. Table 3 shows the results. The null position denotes that the processing time for this step is too small to be measured by the computer. Notice that Table 3 is a composition of steps for the compared algorithms. The total processing time for each algorithm is a sum of some steps. For example, the time for global spin image (GSI) is the sum of “Normal Estimation”, “PCA”, and “GSI”. Table 4 shows the total processing time comparisons, where we also provided the percentage of the normal estimation step to the total processing time.

The results in Table 3 and Table 4 imply that for the 10 proposed shape measures, 3D shape measures take longer time than 2D shape measures. Half of the 10 measures have processing times that are too small and can be ignored. The percentage p shows that the normal estimation step takes the majority portion of the total processing time among each algorithm.

#### 4.4.2. Classifier Training and Testing Phase

We measured the training and testing time of the SVM classifier on all the available 14,631 data samples. Both the linear SVM and Gaussian kernel SVM were used. In each trial, we randomly split the data into training and testing set (approximately 80% for training and 20% for testing) as was done in Section 4.1, Section 4.2 and Section 4.3. The results are averaged over 10 trials, which have been shown in Table 5.

One can observe that the proposed algorithm takes the shortest time in both linear and kernel SVMs. The advantage becomes obvious when compared to GSI or SD due to the fact that the proposed descriptor has only 10 dimensions while GSI and SD have relatively high dimensions. One should notice that SD has lower dimension than GSI but takes longer training time. This is probably because that most of the entries in GSI are zeros and the vectors of SD are dense.

To summarize, the computational time of the proposed shape descriptor is comparable to the existing methods in the feature extraction phase and outperforms them by a large margin in the classifier training and testing phase, which validates the computational complexity analysis in Section 3.4.

## 5. Conclusions and Future Work

This paper proposed a 3D shape descriptor for object recognition with RGB-D sensors and exploits the object recognition ability of shape features. We proposed 10 shape features representing the global and local shape of an object; five from 2D mask images; and five from 3D point cloud data. These features form a hybrid shape descriptor that does not need to employ a quantization mechanism that aggregates local features into object-level representations. Furthermore, it is a low dimensional feature vector compared to state-of-the-art descriptors. We evaluated the classification performance of the shape descriptor at category and instance recognition with several multi-class classifiers. The proposed descriptor outperforms some global-based shape descriptors by a large margin, and is comparable to some local-based shape descriptors. The results imply that the proposed shape features are strong cues for object recognition and can be combined with other features to boost accuracy.

Our future work will focus on improving the limitations of the proposed descriptor. Some normalization is necessary when combining all the shape measures into the final descriptor. The scale-invariant property needs to be addressed. More experiments on real applications will be conducted.

## Figures and Tables

**Table 1:** Accuracies (in percentage) of four shape descriptors on the RGB-D Object Dataset. SD means shape distribution. GSI means global spin image. SH means shape histogram. ± refers to standard deviation.

Classifier	SD	GSI	SH	Our Work
**Category Recognition**				
NB	35.1 ± 0.03	38.2 ± 0.03	33.4 ± 0.03	54.5 ± 0.01
NN	43.8 ± 0.01	47.0 ± 0.02	42.9 ± 0.01	59.2 ± 0.02
LinSVM	47.4 ± 1.89	53.7 ± 2.42	38.2 ± 1.41	63.2 ± 2.69
kSVM	56.9 ± 2.19	54.3 ± 3.49	46.7 ± 1.29	68.7 ± 3.60
**Instance Recognition**				
NB	19.8	27.9	19.4	36.3
NN	20.1	28.8	18.4	25.2
LinSVM	31.5	37.2	20.0	45.7
kSVM	35.5	35.4	21.0	46.0

**Table 2:** Comparisons to state-of-the-art partial-to-global shape descriptors. Accuracy is in percentage. ± refers to standard deviation.

Shape Features	Category	Instance
KPCA [19]	50.2 ± 2.9	29.5
Spin KDES [19]	64.4 ± 3.1	28.8
SF+LinSVM [10]	53.1 ± 1.7	32.3
SF+Ksvm [10]	64.7 ± 2.2	46.2
Our work	66.3 ± 1.8	45.8

**Table 3:** Computational time comparison of major steps in the feature extraction phase.

Steps	Normal Estimation	PCA	GSI	SD	SH
time (s)	8.6138	0.0291	0.0119	0.0782	0.0034
Steps	Compactness 3D	Symmetry 3D	Global convexity 3D	Local convexity 3D	Smoothness 3D
time (s)	-	0.4034	0.7222	0.4366	-
Steps	Compactness 2D	Symmetry 2D	Global convexity 2D	Uniqueness 2D	Smoothness 2D
time (s)	-	0.0013	0.0145	-	-

**Table 4:** Total computational time comparison. *p* denotes the percentage of the normal estimation step to the total processing time.

Methods	GSI	SD	SH	Our Work
Total time (s)	8.6548	8.7211	8.6463	10.2209
*p* (%)	99.53	98.77	99.62	84.28

**Table 5:** Training and testing time comparison.

**Training (s)**	**GSI (1600D)**	**SD (1024D)**	**SH (100D)**	**Our Work (10D)**
Linear SVM	295.5 ± 6.3	1178.2 ± 18.1	98.0 ± 2.9	25.1 ± 0.2
Kernel SVM	2529.3 ± 16.9	4224.2 ± 20.8	94.4 ± 1.6	31.9 ± 0.2
**Testing (s)**	**GSI (1600D)**	**SD (1024D)**	**SH (100D)**	**Our work (10D)**
Linear SVM	77.9 ± 0.7	84.5 ± 0.6	11.1 ± 0.1	9.4 ± 0.1
Kernel SVM	128.9 ± 0.9	97.5 ± 0.6	13.1 ± 0.1	12.3 ± 0.2
